# Risk for psychiatric and substance use disorders as a function of transitions in Sweden’s public educational system: a national study

**DOI:** 10.1017/S003329172300048X

**Published:** 2023-03-07

**Authors:** Kenneth S. Kendler, Richard S. E. Keefe, Henrik Ohlsson, Jan Sundquist, Kristina Sundquist

**Affiliations:** 1 Virginia Institute for Psychiatric and Behavioral Genetics, Virginia Commonwealth University, Richmond, VA, USA; 2 Department of Psychiatry, Virginia Commonwealth University, Richmond, VA, USA; 3 Department of Psychiatry, Duke University Medical Center, Durham, NC, USA; 4 Center for Primary Health Care Research, Lund University, Malmö, Sweden; 5 Department of Family Medicine and Community Health, Department of Population Health Science and Policy, Icahn School of Medicine at Mount Sinai, NY, New York, USA

**Keywords:** Depression, schizophrenia, drug abuse, alcohol use disorder, educational transitions, Sweden

## Abstract

**Background.:**

To clarify, in a national sample, associations between risk for seven psychiatric and substance use disorders and five key transitions in Sweden’s public educational system.

**Methods.:**

Swedish-born individuals (1972–1995, *N* = 1 997 910) were followed through 12-31-2018, at mean age 34.9. We predicted, from these educational transitions, risk for major depression (MD), obsessive-compulsive disorder (OCD), bipolar disorder (BD), schizophrenia (SZ), anorexia nervosa (AN), alcohol use disorder (AUD), and drug use disorder (DUD), assessed from Swedish national registers, by Cox regression, censoring individuals with onsets ⩽17. We also predicted risk from the deviation of grades from family-genetic expectations (deviation 1) and from changes in grades from ages 16 to 19 (deviation 2).

**Results.:**

We observed four major risk patterns across transitions in our disorders: (i) MD and BD, (ii) OCD and SZ, (iii) AUD and DUD, and (iv) AN. Failing early educational transitions had the greatest impact on risk for OCD and SZ while for other disorders, not progressing from basic to upper high school had the largest effect. Completing vocational *v*. college-prep upper high school was strongly associated with risk for AUD and DUD, had little relation with MD, OCD, BD, and SZ risk, and was protective for AN. Deviation 1 predicted risk most strongly for SZ, AN, and MD. Deviation 2 predicted risk most strongly for SZ, AUD, and DUD.

**Conclusions.:**

The pattern of educational transitions and within family and within person development deviations are strongly and relatively specifically associated with future risk for seven psychiatric and substance-use disorders.

A substantial literature shows a range of associations between school performance in childhood and adolescence and later risk for psychiatric and substance use disorders (Isohanni et al., 1998; Pijnenburg et al., 2021). Most such studies are done in small to modest sized samples or examine only one or a few performance measures (Isohanni et al., 1998; Sourander et al., 2009), and/or only one or a small number of disorders (e.g. Chong et al., 2009; Kessler, Foster, Saunders, & Stang, 1995). Further, most studies have included individuals with onset of illness prior to adulthood, which conflates prediction with cross-sectional association (Chong et al., 2009), or have used early mental health problems as predictors of later educational challenges (McLeod & Fettes, 2007). However, in contrast to educational performance, which is evaluated repeatedly over time in almost all school settings, symptoms of psychological, behavioral, and psychiatric illness are less frequently evaluated in late adolescence, even though this period is known to be a crucial time for life-long psychiatric vulnerability (Kessler et al., 2007).

A good demonstration of the power of cognitive performance predictors of later psychiatric illness has been the set of papers generated from linking the Israeli Draft Board Registry with the National Psychiatric Hospitalization Case Registry (Davidson et al., 1999). These studies have found that cognitive performance difficulties predict a range of later disorders, with some distinctions among them such as a worse cognitive performance in draftees who later went on to develop schizophrenia compared with those who developed non-psychotic bipolar disorder (Reichenberg et al., 2002). However, the pattern of relationship of adolescent cognitive and educational performance with the broad range of later psychiatric diagnosis is poorly understood.

Within many school systems, children are sorted at multiple stages as a function of their school performance. This sorting process represents a large-scale ‘social’ experiment that is the focus of this manuscript. We examine a cohort of ~2 million children born in Sweden from 1972 to 1995 and followed through five key binary educational transitions up through age 19. We then examine the impact of each transition on risk for seven diverse psychiatric and substance use disorders, as assessed using Swedish national registers: major depression (MD), obsessive-compulsive disorder (OCD), bipolar disorder (BD), schizophrenia (SZ), anorexia nervosa (AN), alcohol use disorder (AUD), and drug use disorder (DUD). We are particularly interested in determining the patterns across these educational transitions and the degree to which they are similar or distinctive among the various disorders as well as the degree to which disorder risk is predicted by the deviation of grades from family-genetic expectations (deviation 1) and from changes in grades from ages 16 to 19 (deviation 2).

## Methods

We collected longitudinal information on individuals from Swedish population-based registers with national coverage linking each person’s unique personal identification number which, to preserve confidentiality, was replaced with a serial number by Statistics Sweden. We secured ethical approval for this study from the Regional Ethical Review Board in Lund and no participant consent was required as the analyses were based on secondary data (No. 2008/409 and later amendments). In the database, we included age of first registration for the seven disorders: MD, OCD, BD, SZ, AN, AUD, and DUD, utilizing ICD-8, 9, and 10 codes from primary care, specialist, and hospital registers as well as prescription and criminal registries (see Appendix Tables 1 and 2).

### For descriptive purposes, we examine

**Fig. 1.**
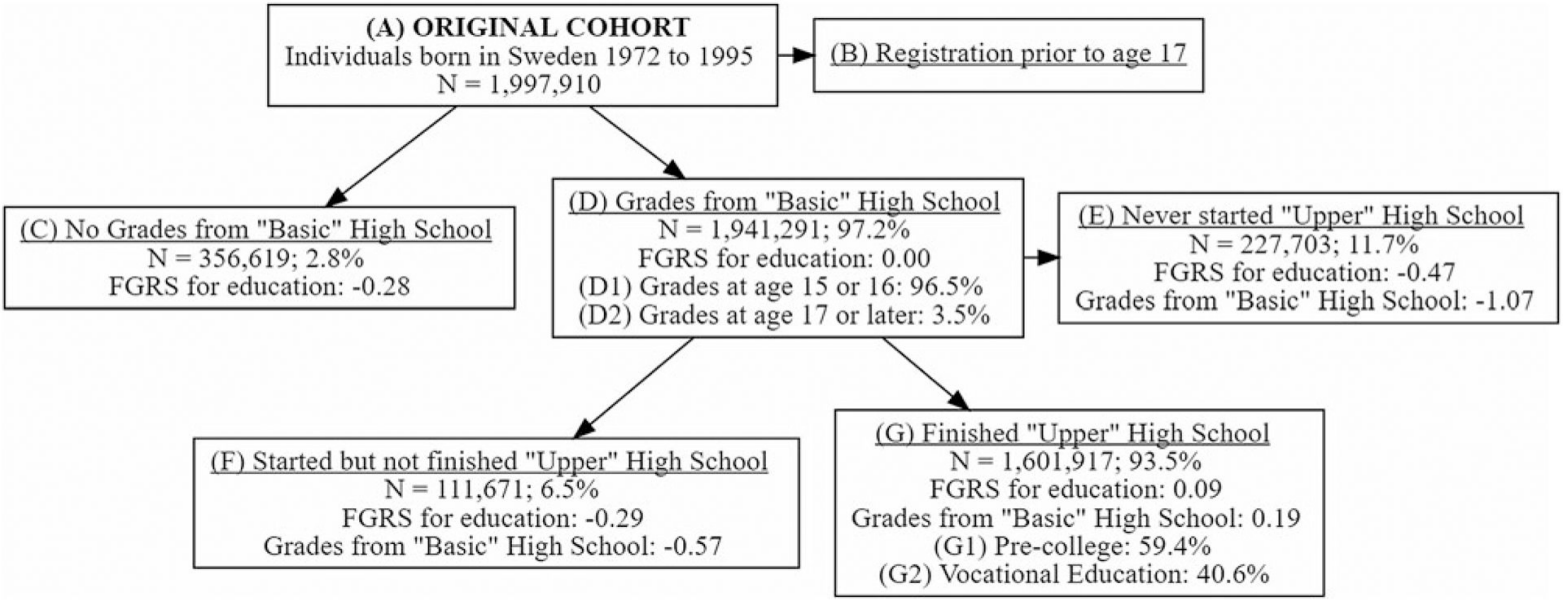
Flow chart of educational transitions of our Swedish study cohort. Starting with the original cohort (*a*), we first censor individuals with a registration for the particular psychiatric disorder being examined prior to age 17. (*b*) Major depression obsessive-compulsive disorder, bipolar disorder, schizophrenia, anorexia nervosa, alcohol use disorder, and drug use disorder were censored from the sample. In each of the subsequent boxes, we give (i) the sample size of subjects, (ii) the % of the relevant group with that outcome and the mean family genetic potential (FGP) for educational attainment as a standardized *z* score. At the next stage, the remaining individuals are divided into those who had no recorded grades for ‘basic’ high school (group C) and those who had recorded grades (group D). Among those in group D, we distinguish between those who completed basic high school on time (ages 15–16 – group D1) and those who were delayed in completing basic high school (group D2). Those in group D then had three possible further educational outcomes. Either they never started upper high school (group E), started but did not complete upper high school (group F) or completed upper high school (group G). We also distinguish those in group G who completed pre-college upper high school (group G1) and those who completed vocational upper high school (group G2).

Based on information from two national school registers, we created seven partially overlapping groups for different educational transitions. The first register contains a grade point average from the compulsory basic high school at the end of grade nine (usually at age 16). The second register contains a grade point average from upper high school (usually at age 18–19), information if an individual started upper high school but did not obtain any grades, and orientation (vocational or pre-college). In Fig. 1, the seven groups are described. The groups are slightly different for each disorder, as we excluded individuals with a registration of the disorder prior to age 17.

For each of the seven disorders, we performed five different Cox regression models. The first model investigated the future risk of a disorder as a function of the first educational transition shown in Fig. 1 [did not *v*. did complete basic high school (C *v*. D) until end of the follow-up (date of disorder, death, emigration, or 12-31-2018], while controlling for year of birth and sex The same model approach was used for the other four educational transitions: (ii) completed basic high school and was delayed *v*. graduated on time (D2 *v*. D1); (iii) completed basic high school and did not *v*. did start upper high school (E *v*. F + G); (iv) started upper high school and did not finish *v*. did finish (F *v*. G); and (v) finished upper vocational high school *v*. pre-college upper high school (G2 *v*. G1).

In the subsample of individuals who completed both basic high school and upper high school (G in Fig. 1), we included the grade point average at age 16, the grade point average at age 19, and based on educational information on first to fifth degree relatives, a familial-genetic potential for educational attainment (FGP_EA_). All three variables were transformed into *Z*-scores with a mean 0 and s.d. 1 (see Appendix Table 2). For each of the seven disorders, we performed a Cox regression model with follow-up to date of disorder, death, emigration, or 12-31-2018 while controlling for year of birth and sex. The main predictor variables were educational achievement at age 16, the deviation of that achievement from the expected level based on the FGP_EA_, and the change in level of educational achievement between ages 16 and 19. All analyses were performed using SAS 9.4 (SAS Institute, 2012).

## Results

### Description of our sample

Our sample included all individuals born in Sweden from 1972 to 1995 to Swedish-born parents and residing in Sweden at age 19 (*N* = 1 997 910). During our study period, all Swedish children go to school for at least 9 years from the year they turn 7, as mandated by the Swedish Education Act. They were all followed up through 12-31-2018, at which point they had a mean (s.d.) age of 34.9 (7.1). Table 1 provides further descriptive statistics of our sample as a function of educational attainment, in particular, the proportion living in deprived and urban areas and coming from a broken home or a home with low parental education (for definition see Appendix Table 1). As expected, on average, subjects who attained higher levels of education tended to be less likely to come from deprived areas or broken homes or to have parents with low educational attainment.

### Educational transitions and risk for illness

In Fig. 1 and Table 2, we outline the sample sizes of the seven partially overlapping groups based upon their levels of education attainment in the national Swedish educational system: (i) did not complete basic high school (group C); (ii) completed basic high school at a typical age (15 or 16) (group D1); (iii) completed basic high school at an older than typical age (⩾17) (group D2); iv) did not start upper high school (group E); (v) started but did not complete upper high school (group F); (vi) finished upper college preparation high school (group G1); and (vii) finished vocational upper high school (group G2). Sample sizes varied substantially from 14 000 who did not finish basic high school to over 230 000 who finished upper high school.

Table 1 also contains the prevalence rates for the seven disorders under consideration. They ranged widely from 0.2% for SZ to 17.6% for MD. Table 1 also notes the proportion of all those cases censored from our analyses due to a first diagnostic registration prior to age 17, which ranged from 7.2% for SZ to 56.0% for AN.

**Fig. 2.**
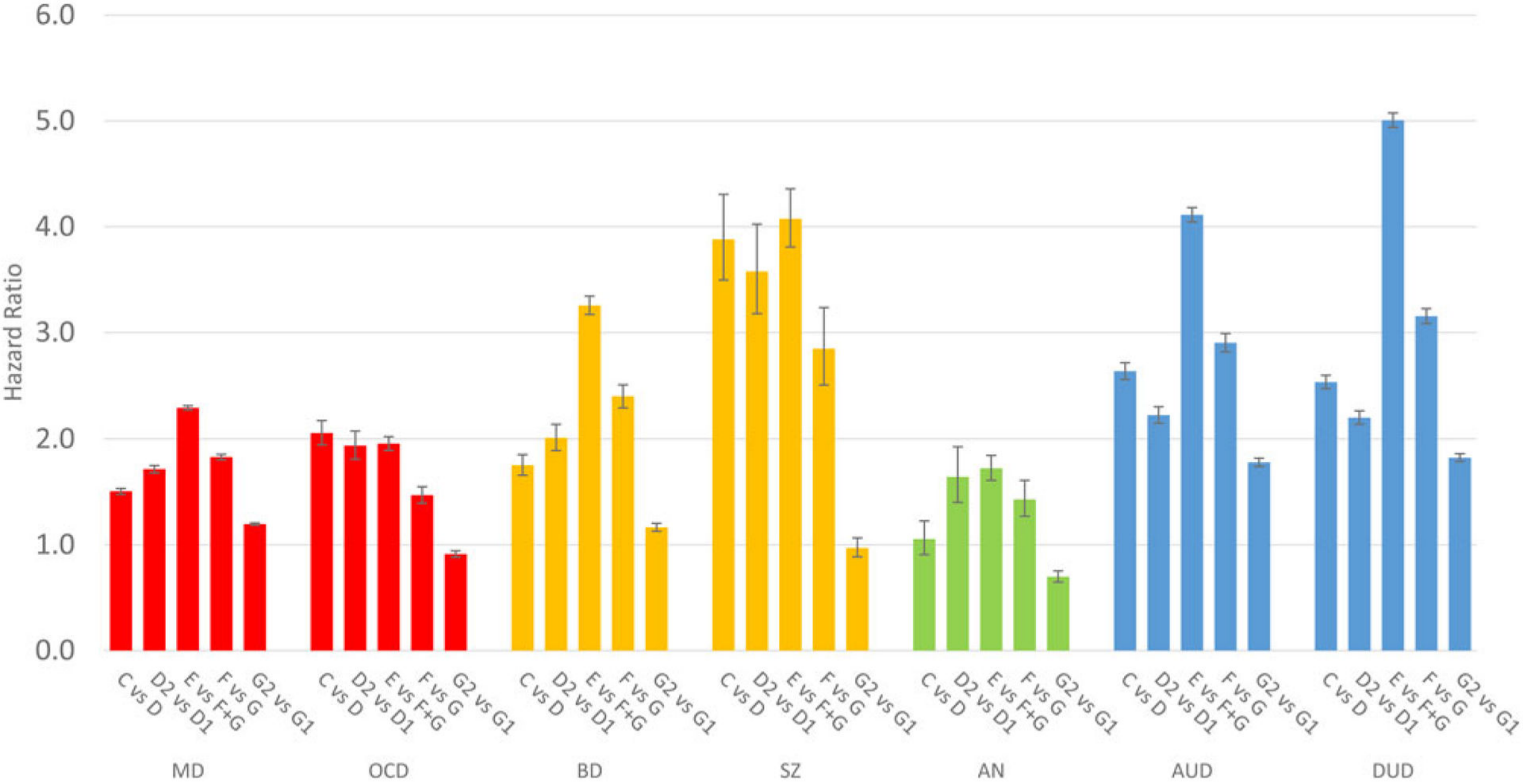
The relationship between five distinct educational transitions and risk for major depression (MD), obsessive-compulsive disorder (OCD), bipolar disorder (BD), schizophrenia (SZ), anorexia nervosa (AN), alcohol use disorder (AUD), and drug use disorder (DUD). The *y*-axis represents the hazard ratio for the specific disorder (±95% confidence intervals) associated with each transition, as calculated by a Cox regression model. See our Methods section for details. For the inter-pretation of each transition, see our Methods section and/or Fig. 1. For example, results for MD for the first transition ‘C *v.* D’ reflects the hazard ratio for MD in individuals who did not *v.* did complete basic high school (C *v.* D in Fig. 1).

Figure 2 depicts the hazard ratios for the five key educational transitions that we examined: (i) did not *v*. did complete basic high school (C *v*. D); (ii) completed basic high school and was delayed *v*. graduated on time (D2 *v*. D1); (iii) completed basic high school and did not *v*. did start upper high school (E *v*. F + G); (iv) started upper high school and did not finish *v*. did finish (F *v*. G); and (v) finished upper vocational high school *v*. college-prep upper high school (G2 *v*. G1). The precise results depicted in Fig. 2 are presented in Appendix Table 3.

The results seen in Fig. 2 reflect striking differences within and between the seven disorders considered. The general HRs associated with the educational transitions were highest for DUD, AUD, and SZ, intermediate for BD, low for MD and OCD, and lowest for AN.

When observing the educational transition differences within disorders, four patterns of risk were evident. First, for both MD and BD, the risk rose monotonically across the first three transitions, peaking at the third transition, not progressing *v*. progressing from basic to upper high school. The HR then declined for the last two transitions with the final transition – vocational *v*. pre-college upper high school – having a HR of 1.2.

The second pattern, seen for both OCD and SZ, presented risks that were at their highest and very similar across the first three transitions, declining substantially with the fourth and fifth transitions. The final transition did not differ from unity for SZ and was slightly below unity for OCD.

The third pattern of risks, seen for AUD and DUD, was characterized by the HR falling from the first to second transition, rising dramatically for the third transition, and then decreasing again for the fourth and fifth transitions. Unlike the first and second patterns, for these two disorders, the final transition was associated with a relatively robust HR of ~1.8.

The fourth pattern was unique to AN. It resembled the pattern seen for MD and BD with one striking difference. The fifth transition was associated with a strong protective effect – that is higher rates of AN were seen in those enrolled in the pre-college *v*. vocational upper high school.

In Appendix Fig. 1, we examine differences in the results presented in Fig. 2 in males and females. Of the 35 contrasts, using a nominal *p* value of 0.01, 17 were statistically significant, clearly above chance expectations. Of note, all six differences seen for MD and OCD were significantly stronger for females while all seven differences seen in AUD and DUD were significantly stronger in males. BD and SZ together had five differences, four stronger in males and one in females.

### Prediction of risk of illness from educational achievement at age 16, deviation from family-genetic expectations and development changes from ages 16–19

**Fig. 3.**
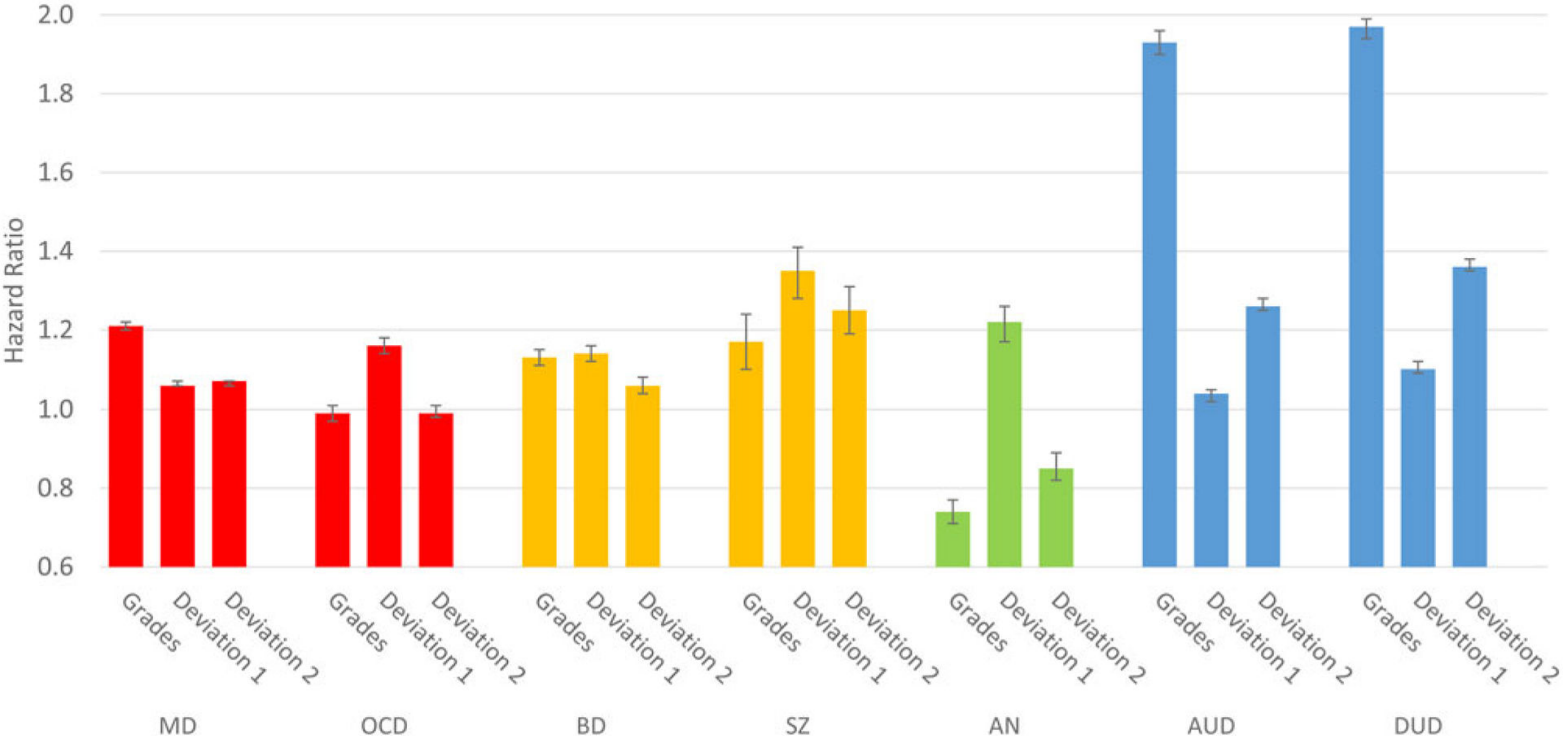
The prediction of risk for major depression (MD), obsessive-compulsive disorder (OCD), bipolar disorder (BD), schizophrenia (SZ), anorexia nervosa (AN), alcohol use disorder (AUD), and drug use disorder (DUD) from three variables in a multivariate cox regression: (i) average school grades at age 17 (grades), (ii) the deviation of those grades from family-genetic expectations for educational attainment (deviation 1), and (ii) the changes in grades from ages 17 to 19 (deviation 2). Family-genetic expectations educational attainment was calculated from first to fifth degree relatives using the familial-genetic potential for educational attainment (FGP_EA_), detailed in the Appendix Table 2. See Methods for further details.

Figure 3 presents the HRs for the seven disorders from a multi-variable model with three predictors: (i) educational achievement at age 16, (ii) the deviation of that achievement from the expected level based on the FGP_EA_, and (iii) the change in level of educational achievement between ages 16 and 19. These analyses could only be conducted in the subsample of individuals who had completed upper high school, thereby eliminating a substantial proportion of those at highest absolute risks in the population. The precise results depicted in Fig. 3 are presented in Appendix Table 4.

The patterns observed across our seven disorders were quite variable. AUD and DUD were distinctive and similar with substantial HRs associated with poor educational achievement at age 16, very little risk resulting from falling short of family-genetic expectations, and a moderate risk for those whose educational achievement declined from ages 16 to 19. SZ and OCD resembled one another in one important way – the strongest risk factor was deviation from the educational expectations based on their FGP_EA_. However, they differed in that grades at age 16 and the change in grades from 16 to 19 were unrelated to risk for OCD but impacted substantially on risk for SZ, as declining grades in SZ was the strongest predictor of all, outside the substance use disorders. All risks were quite modest for MD and BD. For MD, low educational attainment was the strongest risk while for BD, educational attainment and the deviation from family-genetic expectations were of similar modest effect. Results from AN were again unique. Low educational attainment was protective for AN as was doing more poorly in school at age 19 compared to 16. Deviating downward from family-genetic expectations, however, increased risk modestly.

## Discussion

The current study combined educational, medical, and criminal registries in Sweden to include almost 2 million young people with data on individual educational attainment and longitudinal academic performance, family-genetic potential for educational achievement (FGP_EA_), and registrations for a range of psychiatric and substance use disorders in adulthood. FGP_EA_, change over time from 16 to 19 years of age in school performance, and individual educational transitions all predicted later registration for a psychiatric diagnosis, in most cases independently. The pattern of findings differed robustly across diagnostic groups, with four separate patterns of educational transition-based risk observed. These results support and extend previous findings and facilitate additional conclusions about the strong associations of familial and educational measures with later psychiatric and substance use diagnoses. To our knowledge, this is the first study to use FGP_EA_, cross-sectional educational attainment and performance, and longitudinal change in individual educational performance as predictors of later diagnosis. These variables all contributed differently to the distinction between diagnostic groups and support the value of evaluating all of these risk factors independently in each disorder. Space limitations preclude a complete consideration of the complex pattern of results, but the most striking findings are considered below.

Among the educational transitions, the most powerful comparison was typically between those who did not attend upper high school, who had far more registrations of all later adult disorders compared to those who went on to vocational or pre-college upper high school. The smallest difference was between those who completed vocational *v*. pre-college upper high school, even though students who attended vocational school did not perform as well academically. These results suggest that successfully completing any upper high school was protective compared to not attending at all.

Deviation from FGP_EA_ was also a robust predictor of later diagnosis, which we have reported on previously (Kendler, Ohlsson, Mezuk, Sundquist, & Sundquist, 2016a). The power of deviation from FGP_EA_ was particularly strong – with hazard ratios above 1.2 – in SZ, OCD, BP, and AN. In our previous work (Kendler, Ohlsson, Keefe, Sundquist, & Sundquist, 2018a), based upon a smaller sample that was included in the current database, FGP_EA_ predicted autism spectrum disorders and SZ robustly and OCD and ADHD more modestly.

The time period from age 16 to 19 is a crucial time for social and brain development (Blakemore, 2012) and educational attainment, which is associated with future financial potential (Borghans, Golsteyn, Heckman, & Humphries, 2016; French, Homer, Popovici, & Robins, 2015). Given that this period often shapes the direction that a young adult will follow and has a potential impact on long-term success and happiness, it is surprising that very few studies have investigated the impact of educational changes over this time period on later psychiatric illnesses which emphasizes the importance of the current findings. In the current study, worsening grades from age 16 to 19 were a strong predictor of risk for SZ, AUD, and DUD, and were protective for AN. These findings suggest that a progressive decline in student performance is a cause for heightened concern beyond the standard apprehension regarding loss of education in that it may portend later mental illness. Given that the observation of performance decline in high school can be based upon a data set that is collected repeatedly as a matter of course and is immediately available, it offers a cost-effective way of identifying young people who may benefit most from additional services and treatment.

The robust prediction of SZ with all four predictors is novel. Students who would later develop SZ performed more poorly at age 16, their performance worsened from age 16 to 19, they performed more poorly than the expectations set by the cognitive performance of their family members as a whole, and they were more likely to fail to make the educational transitions we evaluated. While multiple studies have demonstrated the value of early cognitive decline (Reichenberg et al., 2010), educational performance (Bilder et al., 2006; Fuller et al., 2002), and IQ (Schulz, Sundin, Leask, & Done, 2014) in predicting SZ, a decline in performance during late adolescence has not been established in large population-based samples such as this one. In fact, some previous studies have suggested that change over time in full-scale IQ did not predict later SZ (Khandaker, Barnett, White, & Jones, 2011). However, other results using clinic-based cognitive performance changes over time in young people at risk for psychosis in much smaller samples (Lam et al., 2018) have suggested that cross-sectional and longitudinal differences are independently predictive of later psychosis. Together, these results offer promise for a more refined prediction of SZ onset (Worthington & Cannon, 2021) with orthogonal contributions from measures of future patient performance at multiple time points and their relation to other sources, particularly family cognitive ability (Kendler et al., 2016a, 2016b; Kendler et al., 2018a). Further, these data conflict with previous studies (Reichenberg et al., 2005; Schulz et al., 2014) that have suggested that only the lowest levels of IQ and cognitive performance predict later SZ. In our sample, it is likely that very low-IQ individuals would never have passed on to upper education levels and so were not represented in those analyses. Yet our data suggest that even at the higher levels of educational transition, those who will later develop SZ perform more poorly and are likely to be transitioned to a lower level of education as a result.

The strongest predictor among those we studied was grade performance in students who would later develop drug or alcohol use disorder. Students who never started secondary school were at 4–5 times greater risk for developing one of the use disorders compared to those who enrolled in vocational or pre-college secondary schools. Those who enrolled in vocational school were at almost two times greater risk compared with those who entered a pre-college program. Further, overall grade performance was associated with a HR greater than 2, and declining grades from 16 to 19 years of age added further risk. While our methodology excluded those participants, who developed diagnosable conditions prior to age 17, it is possible that some young people were abusing substances sufficiently to impair school performance without being registered for a substance use disorder either via the medical or criminal registries diagnosis. Thus, the predictive power of these educational factors may be biased upward by the inclusion in our sample of young people who were abusing drugs or alcohol enough to impair their school performance without a formal diagnosis prior to age 17. However, previous analyses using statistical approaches that infer causality on a version of this Swedish database (Kendler et al., 2018a) have suggested that the direction of causality runs from low early academic achievement in high school to later drug abuse.

The predictive value of educational transition was very different across disorders. While it was quite robust in several of the disorders and transitions, for AN it was the smallest at each transition comparison. Strikingly, only in the AN group was the transition to vocational school *v*. pre-college associated with substantially reduced risk. Further, only in AN was *better* educational performance associated with a *higher* risk of developing a disorder. The AN group was also the only one in which grades and deviation from family-genetic cognitive estimates were predictive in opposite directions, with an increase in adult AN registration predicted by *better* grades and *worse* cognitive performance compared to family-genetic expectations. Given the presence of increased anxiety in children who later develop eating disorders (Raney et al., 2008) and the power of perceived familial pressure in anorexia (Tozzi, Sullivan, Fear, McKenzie, & Bulik, 2003), the data in our study may point toward the possibility that a less stressful academic environment and approach may be protective against AN but that deviation from family-genetic expectations is still a potential stressor. However, it is also reasonable to speculate that social pressures for thinness may have been stronger in the pre-college than vocational upper high schools. These results are consistent with models of stress and perfectionism in educational performance in young people who have developed or will develop future anorexia, particularly those whose academic aspirations exceed their intellectual abilities (Schilder, Sternheim, Aarts, van Elburg, & Danner, 2021).

A wealth of literature has compared the cognitive profiles and history of BD to that of SZ and MD. Patients with BD tend to have cognitive impairment profiles that are slightly more severe than patients with MD but far less severe than patients with SZ (Reichenberg et al., 2009). Previous prospective studies in Israel (Reichenberg et al., 2002), the Netherlands (Vonk et al., 2012), and England (Schulz et al., 2014) that have predicted SZ from premorbid cognitive profiles have failed to predict BD. This study supports these findings, as the educational attainment patterns in BD resemble that of MD much more than that of SZ, yet is inconsistent with molecular genetic results where the genetic correlation of BD is considerably higher with SZ than with MD (Cross-Disorder Group of the Psychiatric Genomics Consortium, 2019).

An additional pattern worth noting is that in the profiles in Figs 2 and 3, OCD resembles, in a more muted form, the pattern seen for SZ in terms of educational transition and deviation from FGP_EA_ and is thereby differentiated from the other five disorders. In both OCD and SZ, not attending high school at all had considerable relative predictive value. Similar findings from the Dunedin Multidisciplinary Health and Developmental study suggested premorbid cognitive deficits in young people who later develop OCD (Grisham, Anderson, Poulton, Moffitt, & Andrews, 2009) or SZ (Reichenberg et al., 2010). It is possible that for both disorders, early cognitive and/or social difficulties lead to the termination of educational involvement as well as adult onset of full blown disorders.

### Limitations

These results should be interpreted in the context of six potential limitations. First, these data reflect the risk for psychiatric disorders in young people in Sweden. The generalization of these data to other countries, particularly those with very different educational systems, may be limited. Second, the validity of our analyses is dependent on the quality of diagnoses in the Swedish registries. This has been studied and supported for the Swedish medical registries in general (Ludvigsson et al., 2011). The validity of MD diagnoses is supported by its prevalence, sex ratio, sibling and twin correlations, and associations with well-documented psychosocial risk factors (Kendler, Ohlsson, Lichtenstein, Sundquist, & Sundquist, 2018b; Sundquist, Ohlsson, Sundquist, & Kendler, 2017) and specific studies have supported the validity of diagnoses for SZ, BD, OCD, and AN (Birgegård et al., 2022; Ekholm et al., 2005; Lichtenstein et al., 2006; Rück et al., 2015; Sellgren, Landen, Lichtenstein, Hultman, & Langstrom, 2011). The validity of our definitions of AUD and DUD are reinforced by the high rates of concordance for registration observed across our ascertainment methods (Kendler et al., 2012, 2015), and the similarity of genetic epidemiological findings for AUD and DUD in Sweden compared to those in other samples (Kendler, Maes, Sundquist, Ohlsson, & Sundquist, 2013; Kendler et al., 2012, 2015, 2016b).

Third, the findings reported in this study suggest associations and do not reveal in any definitive manner the direction of causality. For example, while the developmental course of SZ might suggest that these educational attainment predictors precede the onset of illness, in drug and alcohol use disorders, subclinical abuse symptoms, or a full syndrome that had not yet come to medical attention or produced a criminal record, an undetected disorder may be the cause of the educational difficulties. Our uncertainty about the direction of causation in these analyses illustrates our lack of knowledge about what features of social, psychological, and/or cognitive development precede overt signs of major psychiatric illness. Is it possible that failed educational transitions might serve, among vulnerable populations, as one additional set of rough indicators of possible illness? Further, it is possible for any of the disorders that the young people in the study had not been observed sufficiently to be registered with a diagnosis before the age of 17. To the extent that this occurred, our analyses point to the association of grades and child disorders, and not their predictive value for adult disorders.

Fourth, on average, our cohort was in their mid-30s at the end of follow-up, and we would likely have missed a small number of later age at onset cases of our disorders. Fifth, for practical reasons relating to sample size and availability of data, we confined our analyses to native-born Swedes of Swedish parents, so our results do not necessarily apply to immigrant populations in Sweden.

Sixth, it is worthwhile to obtain another perspective – beyond our use of the hazard ratio – on the value of educational transitions in predicting risk of illness. In Appendix Figs 2 and 3, we show the positive and negative predictive values (PPV and NPV) of each of our educational transitions at predicting our seven disorders. As can be seen, the values of the observed PPVs are markedly affected by prevalence, being highest in MD and lowest for SZ and AN. None are close to high enough to be of clinical utility. The NPVs present the mirror picture.

## Conclusions

In this study of almost 2 million young people in Sweden, sorting young individuals by school performance reveals a robust relationship with risk for a range of psychiatric disorders. These results suggest that familial-genetic cognitive measures and educational measures collected on the basis of school performance and placement predict later psychiatric and behavioral disorders with a set of diagnosis-specific patterns and ranges of severity. The use of these measures in further research is warranted in school settings to facilitate the identification of individuals and groups of young individuals that are at risk for specific psychiatric and substance use disorders.

## Supplementary Material

Supplement 1

## Figures and Tables

**Table 1.** Demographic features of our sample

	(A) Original cohort	(C) Individuals who did not finish high school	(D) Grades from basic high school	(E) Never started upper high school	(F) Started but not finished upper high school	(G) Finished upper high school
*N*	1 997 910	56 619	1 941 291	227 703	111 671	1 601 917
Deprived area (%)	10.1%	16.9%	9.9%	15.7%	12.1%	8.9%
Urban area (%)	64.2%	66.3%	64.1%	64.4%	63.6%	64.1%
Low parental education (%)	12.4%	17.5%	12.3%	20.7%	8.6%	11.4%
Not stable home	31.4%	51.0%	30.8%	49.4%	46.2%	27.1%

**Table 2.** Descriptive statistics for the different samples

	(A) Total number of registrations	(B) Prior to age 17	(C) Individuals who did not finish high school	(E) Individuals who did not start upper secondary school	(F) Individuals who started upper secondary school but not finished	(G) Individuals who started upper secondary school and finished	G1 – Pre-college	G2 – Vocational education^d^
MD	351 760^a^ (17.6%)	32 870^b^ (9.3%)	14 013 (4.0–24.7%)	71 836^c^ (20.4–31.5%)	28 328^c^ (8.1–25.4%)	237 583^c^ (67.5–14.8%)	129 515^c^ (36.8–14.1%)	102 775^c^ (29.2–15.8%)
OCD	25 408 (1.3%)	3863 (15.2%)	1528 (6.0–2.7%)	4799 (18.9–2.1%)	1948 (7.7–1.7%)	17 133 (67.4–1.1%)	10 231 (40.3–1.1%)	6469 (25.5–1.0%)
BD	29 168 (1.5%)	2228 (7.6%)	1451 (5.0–2.6%)	8263 (28.3–3.6%)	2712 (9.3–2.4%)	16 742 (57.4–1.0%)	9175 (31.5–1.0%)	7165 (24.6–1.1%)
SZ	4196 (0.2%)	303 (7.2%)	434 (10.3–0.8%)	1418 (33.8–0.6%)	331 (7.9–0.3%)	2013 (48.0–0.1%)	1154 (27.5–0.1%)	801 (19.1–0.1%)
AN	9734 (0.5%)	5449 (56.0%)	318 (3.3–0.6%)	1570 (16.1–0.7%)	795 (8.2–0.7%)	7051 (72.4–0.4%)	4841 (49.7–0.5%)	2047 (21.0–0.3%)
AUD	76 686 (3.8%)	17 823 (23.2%)	5344 (7.0–9.4%)	25 183 (32.8–11.1%)	6992 (12.4–6.3%)	39 167 (51.1–2.4%)	16 730 (21.8–1.8%)	21 458 (28.0–3.3%)
DUD	109 779 (5.5%)	25 981 (23.7%)	8421 (7.7–14.9%)	37 851 (34.5–16.6%)	13 656 (12.3–12.2%)	49 851 (45.4–3.1%)	19 467 (17.7–2.1%)	28 922 (19.5–4.5%)

a The percentage value shows the prevalence in the population.

b The percentage value shows the share of registrations for the disorder that were registered prior to age 17.

c The first percentage value shows the share of registrations for the disorder that were registered in the specific population; the second percentage value shows the prevalence in the specific population.

d Some individuals could not be categorized as pre-college or vocational education.

## References

[R1] Bilder RM, Reiter G, Bates J, Lencz T, Szeszko P, Goldman RS, … Kane JM (2006). Cognitive development in schizophrenia: Follow-back from the first episode. Journal of Clinical and Experimental Neuropsychology, 28(2), 270–282. 16484098 10.1080/13803390500360554

[R2] Birgegård A, Mantilla EF, Dinkler L, Hedlund E, Savva A, Larsson H, & Bulik CM (2022). Validity of eating disorder diagnoses in the Swedish national patient register. Journal of Psychiatric Research, 150, 227–230. 35398665 10.1016/j.jpsychires.2022.03.064

[R3] Blakemore S-J (2012). Development of the social brain in adolescence. Journal of the Royal Society of Medicine, 105(3), 111–116. 22434810 10.1258/jrsm.2011.110221 PMC3308644

[R4] Borghans L, Golsteyn BH, Heckman JJ, & Humphries JE (2016). What grades and achievement tests measure. Proceedings of the National Academy of Sciences, 113(47), 13354–13359. 10.1073/pnas.1601135113 PMC5127298 27830648

[R5] Chong SA, Subramaniam M, Lee I-M, Pek E, Cheok C, Verma S, & Wong J (2009). Academic attainment: A predictor of psychiatric disorders? Social Psychiatry and Psychiatric Epidemiology, 44(11), 999–1004. 19294321 10.1007/s00127-009-0027-3

[R6] Cross-Disorder Group of the Psychiatric Genomics Consortium. (2019). Genomic relationships, novel loci, and pleiotropic mechanisms across eight psychiatric disorders. Cell, 179(7), 1469–1482.e1411. doi: 10.1016/j.cell.2019.11.020 31835028 PMC7077032

[R7] Davidson M, Reichenberg A, Rabinowitz J, Weiser M, Kaplan Z, & Mark M (1999). Behavioral and intellectual markers for schizophrenia in apparently healthy male adolescents. American Journal of Psychiatry, 156(9), 1328–1335. doi: 10.1176/ajp.156.9.1328 10484941

[R8] Ekholm B, Ekholm A, Adolfsson R, Vares M, Osby U, Sedvall GC, & Jonsson EG (2005). Evaluation of diagnostic procedures in Swedish patients with schizophrenia and related psychoses. Nordic Journal of Psychiatry, 59(1), 457–464. 16316898 10.1080/08039480500360906

[R9] French MT, Homer JF, Popovici I, & Robins PK (2015). What you do in high school matters: High school GPA, educational attainment, and labor market earnings as a young adult. Eastern Economic Journal, 41(3), 370–386.

[R10] Fuller R, Nopoulos P, Arndt S, O’Leary D, Ho BC, & Andreasen NC (2002). Longitudinal assessment of premorbid cognitive functioning in patients with schizophrenia through examination of standardized scholastic test performance. American Journal of Psychiatry, 159(7), 1183–1189. doi: 10.1176/appi.ajp.159.7.1183 12091197

[R11] Grisham JR, Anderson TM, Poulton R, Moffitt TE, & Andrews G (2009). Childhood neuropsychological deficits associated with adult obsessive–compulsive disorder. The British Journal of Psychiatry, 195(2), 138–141. 19648544 10.1192/bjp.bp.108.056812 PMC2801824

[R12] Isohanni I, Järvelin MR, Nieminen P, Jones P, Rantakallio P, Jokelainen J, & Isohanni M (1998). School performance as a predictor of psychiatric hospitalization in adult life. A 28–year follow-up in the Northern Finland 1966 Birth Cohort. Psychological Medicine, 28(4), 967–974. 9723151 10.1017/s0033291798006928

[R13] Kendler KS, Ji J, Edwards AC, Ohlsson H, Sundquist J, & Sundquist K (2015). An extended Swedish national adoption study of alcohol use disorder. JAMA Psychiatry, 72(3), 211–218. doi: 2088151 [pii]; 10.1001/jamapsychiatry.2014.2138 [doi]. 25565339 PMC4351126

[R14] Kendler KS, Maes HH, Sundquist K, Ohlsson H, & Sundquist J (2013). Genetic and family and community environmental effects on drug abuse in adolescence: A Swedish national twin and sibling study. American Journal of Psychiatry, 171(2), 209–217. 10.1176/appi.ajp.2013.12101300 PMC3927993 24077613

[R15] Kendler KS, Ohlsson H, Keefe RSE, Sundquist K, & Sundquist J (2018a). The joint impact of cognitive performance in adolescence and familial cognitive aptitude on risk for major psychiatric disorders: A delineation of four potential pathways to illness. Molecular Psychiatry, 23(4), 1076–1083. doi: 10.1038/mp.2017.78 28416810 PMC5647225

[R16] Kendler KS, Ohlsson H, Lichtenstein P, Sundquist J, & Sundquist K (2018b). The genetic epidemiology of treated major depression in Sweden. American Journal of Psychiatry, 175(11), 1137–1144. doi: 10.1176/appi.ajp.2018.17111251 30021458

[R17] Kendler KS, Ohlsson H, Mezuk B, Sundquist JO, & Sundquist K (2016a). Observed cognitive performance and deviation from familial cognitive aptitude at age 16 years and ages 18 to 20 years and risk for schizophrenia and bipolar illness in a Swedish national sample. JAMA Psychiatry, 73(5), 465–471. doi: 2504224 [pii]; 10.1001/jamapsychiatry.2016.0053 [doi]. 27028264

[R18] Kendler KS, PirouziFard M, Lonn S, Edwards AC, Maes HH, Lichtenstein P, … Sundquist K (2016b). A national Swedish twin-sibling study of alcohol use disorders. Twin Research and Human Genetics, 19(5), 430–437. doi: S1832427416000621 [pii]; 10.1017/thg.2016.62 [doi]. 27515133 PMC5064148

[R19] Kendler KS, Sundquist K, Ohlsson H, Palmer K, Maes H, Winkleby MA, & Sundquist J (2012). Genetic and familial environmental influences on the risk for drug abuse: A national Swedish adoption study. Archive of General Psychiatry, 69(7), 690–697. doi: 10.1001/archgenpsychiatry.2011.2112 PMC3556483 22393206

[R20] Kessler RC, Amminger GP, Aguilar-Gaxiola S, Alonso J, Lee S, & Ustun TB (2007). Age of onset of mental disorders: A review of recent literature. Current Opinion in Psychiatry, 20(4), 359. 17551351 10.1097/YCO.0b013e32816ebc8c PMC1925038

[R21] Kessler RC, Foster CL, Saunders WB, & Stang PE (1995). Social consequences of psychiatric disorders, I: Educational attainment. American Journal of Psychiatry, 152(7), 1026–1032. 7793438 10.1176/ajp.152.7.1026

[R22] Khandaker GM, Barnett JH, White IR, & Jones PB (2011). A quantitative meta-analysis of population-based studies of premorbid intelligence and schizophrenia. Schizophrenia Research, 132(2–3), 220–227. doi: S0920-9964(11)00322-7 [pii]; 10.1016/j.schres.2011.06.017 [doi]. 21764562 PMC3485562

[R23] Lam M, Lee J, Rapisarda A, See YM, Yang Z, Lee SA, … Keefe RSE (2018). Longitudinal cognitive changes in young individuals at ultrahigh risk for psychosis. JAMA Psychiatry, 75(9), 929–939. doi: 10.1001/jamapsychiatry.2018.1668 30046827 PMC6142925

[R24] Lichtenstein P, Bjork C, Hultman CM, Scolnick E, Sklar P, & Sullivan PF (2006). Recurrence risks for schizophrenia in a Swedish national cohort. Psychological Medicine, 36(10), 1417–1425. Retrieved from http://www.ncbi.nlm.nih.gov/pubmed/16863597. 16863597 10.1017/S0033291706008385

[R25] Ludvigsson JF, Andersson E, Ekbom A, Feychting M, Kim JL, Reuterwall C, … Olausson PO (2011). External review and validation of the Swedish national inpatient register. BMC Public Health, 11, 450. doi: 1471-2458-11-450 [pii]; 10.1186/1471-2458-11-450 [doi]. 21658213 PMC3142234

[R26] McLeod JD, & Fettes DL (2007). Trajectories of failure: The educational careers of children with mental health problems. American Journal of Sociology, 113(3), 653–701. 10.1086/521849 PMC2766187 19855855

[R27] Pijnenburg LJ, de Haan L, Smith L, Rabinowitz J, Levine SZ, Reichenberg A, & Velthorst E (2021). Early predictors of mental health in mid-adulthood. Early Intervention in Psychiatry, 15(1), 158–166. 31943798 10.1111/eip.12924

[R28] Raney T, Thornton LM, Berrettini W, Brandt H, Crawford S, Fichter MM, … LaVia M (2008). Influence of overanxious disorder of childhood on the expression of anorexia nervosa. International Journal of Eating Disorders, 41(4), 326–332. 18213688 10.1002/eat.20508 PMC8048416

[R29] Reichenberg A, Caspi A, Harrington H, Houts R, Keefe RS, Murray RM, … Moffitt TE (2010). Static and dynamic cognitive deficits in childhood preceding adult schizophrenia: A 30-year study. The American Journal of Psychiatry, 167(2), 160–169. doi: appi.ajp.2009.09040574 [pii]; 10.1176/appi.ajp.2009.09040574 [doi]. 20048021 PMC3552325

[R30] Reichenberg A, Harvey PD, Bowie CR, Mojtabai R, Rabinowitz J, Heaton RK, & Bromet E (2009). Neuropsychological function and dys-function in schizophrenia and psychotic affective disorders. Schizophrenia Bulletin, 35(5), 1022–1029. doi: 10.1093/schbul/sbn044 18495643 PMC2728814

[R31] Reichenberg A, Weiser M, Rabinowitz J, Caspi A, Schmeidler J, Mark M, … Davidson M (2002). A population-based cohort study of premorbid intellectual, language, and behavioral functioning in patients with schizophrenia, schizoaffective disorder, and nonpsychotic bipolar disorder. American Journal of Psychiatry, 159(12), 2027–2035. 12450952 10.1176/appi.ajp.159.12.2027

[R32] Reichenberg A, Weiser M, Rapp MA, Rabinowitz J, Caspi A, Schmeidler J, … Davidson M (2005). Elaboration on premorbid intellectual performance in schizophrenia: Premorbid intellectual decline and risk for schizophrenia. Archives of General Psychiatry, 62(12), 1297–1304. doi: 10.1001/archpsyc.62.12.1297 16330717

[R33] Rück C, Larsson KJ, Lind K, Perez-Vigil A, Isomura K, Sariaslan A, … Mataix-Cols D (2015). Validity and reliability of chronic tic disorder and obsessive-compulsive disorder diagnoses in the Swedish National Patient Register. BMJ Open, 5(6), e007520. 10.1136/bmjopen-2014-007520 PMC4480012 26100027

[R34] SAS Institute, I. (2012). SAS/STAT^®^ online documentation, Version 9.4. Cary, N. C.: SAS Institute, Inc. In. (Reprinted from: Not in File).

[R35] Schilder CMT, Sternheim LC, Aarts E, van Elburg AA, & Danner UN (2021). Relationships between educational achievement, intelligence, and perfectionism in adolescents with eating disorders. International Journal of Eating Disorders, 54(5), 794–801. doi: 10.1002/eat.23482 33554341

[R36] Schulz J, Sundin J, Leask S, & Done DJ (2014). Risk of adult schizophrenia and its relationship to childhood IQ in the 1958 British birth cohort. Schizophrenia Bulletin, 40(1), 143–151. doi: 10.1093/schbul/sbs157 23277615 PMC3885293

[R37] Sellgren C, Landen M, Lichtenstein P, Hultman CM, & Langstrom N (2011). Validity of bipolar disorder hospital discharge diagnoses: File review and multiple register linkage in Sweden. Acta Psychiatrica Scandinavica, 124(6), 447–453. doi: 10.1111/j.1600-0447.2011.01747.x 21838734

[R38] Sourander A, Klomek AB, Niemela S, Haavisto A, Gyllenberg D, Helenius H, … Gould MS (2009). Childhood predictors of completed and severe suicide attempts: Findings from the Finnish 1981 Birth Cohort Study. Archives of General Psychiatry, 66(4), 398–406. doi: 10.1001/archgenpsychiatry.2009.21 19349309

[R39] Sundquist J, Ohlsson H, Sundquist K, & Kendler KS (2017). Common adult psychiatric disorders in Swedish primary care (where most mental health patients are treated). BMC Psychiatry, 17, 1–9. 28666429 10.1186/s12888-017-1381-4 PMC5493066

[R40] Tozzi F, Sullivan PF, Fear JL, McKenzie J, & Bulik CM (2003). Causes and recovery in anorexia nervosa: The patient’s perspective. International Journal of Eating Disorders, 33(2), 143–154. doi: 10.1002/eat.10120 12616580

[R41] Vonk R, Van Der Schot A, Van Baal G, Van Oel C, Nolen W, & Kahn R (2012). Premorbid school performance in twins concordant and discordant for bipolar disorder. Journal of Affective Disorders, 136(3), 294–303. 22166398 10.1016/j.jad.2011.11.034

[R42] Worthington MA, & Cannon TD (2021). Prediction and prevention in the clinical high-risk for psychosis paradigm: A review of the current status and recommendations for future directions of inquiry. Frontiers in psychiatry, 12, 770–774. doi: 10.3389/fpsyt.2021.770774 PMC8569129 34744845

