# Supplementary material for: Risk for psychiatric and substance use disorders as a function of transitions in Sweden’s public educational system: a national study
Source: Psychol Med. Author manuscript; Available in PMC 2024 Mar 6. (PMC10916708; doi:10.1017/S003329172300048X)
Supplement: Supplement 1 [file NIHMS1963855-supplement-Supplement_1.docx]

**Appendix Table 1: Description of Register**

*National Patient Register*

In the 1960's the National Board of Health and Welfare started to collect information regarding in-patients at public hospitals, the National Patient Register (NPR). Initially it contained information about all patients treated in psychiatric care and approximately 16 percent of patients in somatic care. The register at that time covered six of the 26 county councils in Sweden. In 1984, the Ministry of Health and Welfare together with the Federation of County Councils decided a mandatory participation for all county councils. From 1987, NPR includes all in-patient care in Sweden. Since 2001, the register also covers outpatient doctor visits including day surgery and psychiatric care from both private and public caregivers. The National Patient Register has coverage to 2018. For more information, see https://www.socialstyrelsen.se/en/statistics-and-data/registers/register-information/the-national-patient-register/

*Primary Care Registry*

We also used information from our new Primary Care Registry (PCR), a research dataset including individual-level information on clinical diagnoses from primary health care centers from the following 15 of the 21 Swedish counties: Blekinge (2009-2018), Värmland (2005-2018), Kalmar (2007-2018), Sörmland (1997-2018), Uppsala (2005-2018), Västernorrland (2008-2018), Norrbotten (2009-2018), Gävleborg (2010-2018), Halland (2007-2018), Jönköping (2008-2018), Kronoberg (2006-2018), Skåne (1998-2018), Östergötland (1997-2018), Stockholm (2003-2018), and Västergötland (2000-2018). In 2016, these counties included 87% of the Swedish population. For more information see Sundquist, J., Ohlsson, H., Sundquist, K. et al. Common adult psychiatric disorders in Swedish primary care where most mental health patients are treated. BMC Psychiatry 17, 235 (2017).

*Descriptive Social variables*

Deprived area: Residing in a DeSO area (administrative areas in Sweden) defined as deprived at age 15. Deprivation is based on rates of unemployed, social welfare recipients, and low income individuals^1^

Urban area: Measured at age 15 of the individual. Residing in a DeSO area which is located in the municipality's central location.

Low Parental Education: >=9 years of education among parents

Not Stable home: Not residing with both parents during all years during ages 0 to 15

**Appendix Table 2**

**Definition of disorders and variables**

| Variable/Disorder | Registers | Definition |
| --- | --- | --- |
| Major Depression (MD) | The National Patient Register, Primary Care Registry | ICD-8: 296.2, 298.0, 300.4; ICD-9: 296.2, 296.4, 298.0, 300.4; ICD-10: F32, F33.  **Note**: all individuals with a registration for BD were excluded. |
| Obsessive-Compulsive Disorder [OCD] | The National Patient Register, Primary Care Registry | ICD-9: 300D; ICD-10: F42 |
| Bipolar Disorder (BD) | The National Patient Register, Primary Care Registry | ICD-8: 296.1, 296.3, 296.8, 296.9, 298.1; ICD-9: 296A, 296C, 296D, 296E, 296W, 298B; ICD-10: F30, F31 |
| Schizophrenia (SZ) | The National Patient Register, Primary Care Registry | ICD-8: 295.1, 295.2, 2953, 295.9, 295.6; ICD-9: 295B, 295C, 295D, 295G, 295X; ICD-10: F200, F201, F202, F203, F205, F209 |
| Anorexia Nervosa (AN) | The National Patient Register, Primary Care Registry | ICD-9: 307B; ICD-10: F500 |
| Alcohol Use Disorder (AUD) | The National Patient Register, Primary Care Registry; the Swedish Drug Register (2005-2018); the Swedish Mortality Register, and the Swedish Criminal Register (1973-2018) and the Swedish Suspicion Register (1998-2018) | Alcohol Use Disorder (AUD) was identified in the Swedish medical and mortality registries by ICD codes: ICD9: V79B, 305A, 357F, 571A-D, 425F, 535D, 291, 303, 980; ICD 10: E244, G312, G621, G721, I426, K292, K70, K852, K860, O354, T51, F10); in the Crime Register by codes 3005, 3201, which reflect crimes related to alcohol abuse; in the Suspicion Register by codes 0004, 0005 (Only those individuals with at least two alcohol-related crimes or suspicion of crimes from both Crime Register and Suspicion Register were included); in the Prescribed Drug Register by the drugs disulfiram (Anatomical Therapeutic Chemical (ATC) Classification System N07BB01), acamprosate (N07BB03), and naltrexone (N07BB04). |
| Drug Use Disorder (DUD) | The National Patient Register, Primary Care Registry; the Swedish Drug Register (2005-2018); the Swedish Mortality Register, and the Swedish Criminal Register (1973-2018) and the Swedish Suspicion Register (1998-2018) | Drug abuse (DA) was identified in the Swedish medical and mortality registries by ICD codes (ICD8: Drug dependence (304); ICD9: Drug psychoses (292) and Drug dependence (304); ICD10: Mental and behavioral disorders due to psychoactive substance use (F10-F19), except those due to alcohol (F10) or tobacco (F17)); in the Suspicion Register by codes 3070, 5010, 5011, and 5012, that reflect crimes related to DA; and in the Crime Register by references to laws covering narcotics (law 1968:64, paragraph 1, point 6) and drug-related driving offences (law 1951:649, paragraph 4, subsection 2 and paragraph 4A, subsection 2). DA was identified in individuals (excluding those suffering from cancer) in the Prescribed Drug Register who had retrieved (in average) more than four defined daily doses a day for 12 months from either of Hypnotics and Sedatives (Anatomical Therapeutic Chemical (ATC) Classification System N05C and N05BA) or Opioids (ATC: N02A). |
| Educational Achievement at age 16 (Average grade point) | The National School Registry. | Contains educational achievement (a grade point average) for all students at the end of basic high school (grade nine; usually at age 16). Students had an incentive to perform well in this school year because those with high grades were more likely to gain admission to the desirable upper secondary schools. For each year and by gender we standardized the grade score into a Z-score. From 1988 to 1997, the score was expressed on a scale between 1 (lowest) and 5 (overall mean was 3.2), and students were assessed by a peer referencing system. Grades awarded reflected the position of the student within Sweden and a set of correction factors were applied to ensure that the grades were equivalent between schools. Using this system, the grades had minimal grade inflation over time and were normally distributed. From 1998, the score was expressed on scale between 10 (lowest) and 320 (overall mean was 207) utilizing a criterion referenced system, in which students were assessed for their achievement of certain competencies. Scores were not standardized across schools or constructed to produce a normal distribution. |
| Educational Achievement at age 18/19 (Average grade point) | The National School Registry | Contains educational achievement (a grade point average) for all students at the end of upper High School (usually at age 18/19) For each year and by gender we standardized the grade score into a Z-score. From 1988 to 1997, the score was expressed on a scale between 1 (lowest) and 5 (overall mean was 3.2), and students were assessed by a peer referencing system. Grades awarded reflected the position of the student within Sweden and a set of correction factors were applied to ensure that the grades were equivalent between schools. Using this system, the grades had minimal grade inflation over time and were normally distributed. From 1998, the score was expressed on scale between 10 (lowest) and 320 (overall mean was 207) utilizing a criterion referenced system, in which students were assessed for their achievement of certain competencies. Scores were not standardized across schools or constructed to produce a normal distribution. |
| Familial Genetic Potential for Education (FGP_EA_) | The National School Registry, Multigenerational Register, LISA database | For all individuals in our sample, we used the Multigenerational Register to identify all 1^st^ to 5^th^ degree relatives. For these relatives, we used information on Average grade point at age 18/19, Average grade point at age 16 and Number of years of education.  Number of years of education are measured in 7 different levels  1 Pre-high school < 9 years  2 High School 9 years  3 Upper Secondary School < 3 years  4 Upper Secondary School 3 years  5 Post-secondary education < 3years  6 Post-secondary education 3 years or more  7 Research education (PhD).  All three educational variables are standardized with mean 0 and SD 1. For all relatives we took the mean Z-score for future calculations. For parents and siblings we corrected for cohabitation effects. To estimate the cohabitation effect (i.e. “shared environment”), we created a database with all individuals in the Swedish population born in Sweden 1955-1990. We also included the number of years, during ages 0-15, that individuals resided in the same household as their biological father. We thereby were able to define two kinds of families i) “not-lived-with” father families (offspring never resided for more than 1 year in the same household or in the same community as their biological father); ii) “lived-with” father (offspring resided a minimum of 13 year in the same household as their biological father. We performed a linear regression model with the Z-score for education trait in offspring as outcome and the Z-score for education in father, type of father, and their interaction as predictors. We used the interaction term as the difference of effect between genes only and genes + environment. The same approach was performed for half-siblings where we compared those who were reared together versus reared apart. (For parents this component was 0.87 and for siblings it was 0.76)  For each relative we then calculated the product using the three components: mean Z-score, cohabitation effects, proportion of shared genetic effects (0.003125 -0.5) with the proband. Then we average this product across all relatives to a proband. Then we corrected for the number of relatives. We multiplied the results from the previous step with a shrinkage factor. (Shrinkage factor (SF): B/(B+A/C). It produces more shrinkage if B and C are small, and A is large. (A) = the variance of the z-score of the disorder across all relatives, (B) = the variance in the mean z-score across all probands, (C) = the weighted number of relatives for each proband. We standardized the risk score by year of birth and county of the proband into a z-score with mean 0 and SD 1. This was then used as the FGP_EA_ in the analyses. |
| Deviation of educational achievement at age 16 (Average grade point) from the expected level based on the FGRS_EA_. |  | The standardized value for Average grade point at age 16 minus the standardized value for the Familial Genetic Risk Score for education (FGRS_EA_). Positive Z-values suggest that the individual perform better than expected, while negative value perform worse than expected |

**Appendix Table 3**

**The precise results depicted in figure 2**

| **Disorder** | **Educational transition** | **Hazard Ratio** | **95% CI Lower Limit** | **95% CI Upper Limit** |
| --- | --- | --- | --- | --- |
| Major Depression (MD) | **C vs D** | 1.51 | 1.48 | 1.53 |
|  | **D2 vs D1** | 1.72 | 1.68 | 1.75 |
|  | **E vs F+G** | 2.29 | 2.27 | 2.31 |
|  | **F vs G** | 1.83 | 1.80 | 1.85 |
|  | **G2 vs G1** | 1.20 | 1.19 | 1.21 |
|  |  |  |  |  |
| Obsessive-Compulsive Disorder [OCD] | **C vs D** | 2.06 | 1.94 | 2.17 |
|  | **D2 vs D1** | 1.94 | 1.81 | 2.07 |
|  | **E vs F+G** | 1.96 | 1.89 | 2.02 |
|  | **F vs G** | 1.47 | 1.39 | 1.55 |
|  | **G2 vs G1** | 0.91 | 0.88 | 0.94 |
|  |  |  |  |  |
| Bipolar Disorder  (BD) | **C vs D** | 1.75 | 1.66 | 1.85 |
|  | **D2 vs D1** | 2.01 | 1.89 | 2.14 |
|  | **E vs F+G** | 3.26 | 3.17 | 3.34 |
|  | **F vs G** | 2.40 | 2.29 | 2.51 |
|  | **G2 vs G1** | 1.17 | 1.13 | 1.20 |
|  |  |  |  |  |
| Schizophrenia  (SZ) | **C vs D** | 3.88 | 3.50 | 4.31 |
|  | **D2 vs D1** | 3.58 | 3.18 | 4.02 |
|  | **E vs F+G** | 4.07 | 3.81 | 4.36 |
|  | **F vs G** | 2.85 | 2.50 | 3.24 |
|  | **G2 vs G1** | 0.97 | 0.89 | 1.06 |
|  |  |  |  |  |
| Anorexia Nervosa (AN) | **C vs D** | 1.05 | 0.91 | 1.23 |
|  | **D2 vs D1** | 1.64 | 1.40 | 1.93 |
|  | **E vs F+G** | 1.72 | 1.61 | 1.85 |
|  | **F vs G** | 1.43 | 1.27 | 1.61 |
|  | **G2 vs G1** | 0.70 | 0.65 | 0.75 |
|  |  |  |  |  |
| Alcohol Use Disorder (AUD) | **C vs D** | 2.63 | 2.56 | 2.71 |
|  | **D2 vs D1** | 2.23 | 2.15 | 2.31 |
|  | **E vs F+G** | 4.11 | 4.05 | 4.18 |
|  | **F vs G** | 2.90 | 2.82 | 2.99 |
|  | **G2 vs G1** | 1.78 | 1.74 | 1.82 |
|  |  |  |  |  |
| Drug Use Disorder (DUD) | **C vs D** | 2.53 | 2.47 | 2.59 |
|  | **D2 vs D1** | 2.20 | 2.14 | 2.27 |
|  | **E vs F+G** | 5.01 | 4.94 | 5.08 |
|  | **F vs G** | 3.15 | 3.08 | 3.23 |
|  | **G2 vs G1** | 1.82 | 1.79 | 1.86 |

**Appendix Table 4**

**The precise results depicted in figure 3**

Deviation 1 = The deviation of that achievement from the expected level based on the FGRS_EA_

Deviation 2 = The change in level of educational achievement between ages 16-19.

| **Disorder** | **Variable** | **Hazard Ratio** | **95% CI Lower Limit** | **95% CI Upper Limit** |
| --- | --- | --- | --- | --- |
| Major Depression (MD) | Educational achievement at age 16 | 1.21 | 1.2 | 1.22 |
|  | Deviation 1 | 1.06 | 1.06 | 1.07 |
|  | Deviation 2 | 1.07 | 1.06 | 1.07 |
|  |  |  |  |  |
| Obsessive-Compulsive Disorder [OCD] | Educational achievement at age 16 | 0.99 | 0.97 | 1.01 |
|  | Deviation 1 | 1.16 | 1.14 | 1.18 |
|  | Deviation 2 | 0.99 | 0.98 | 1.01 |
|  |  |  |  |  |
| Bipolar Disorder  (BD) | Educational achievement at age 16 | 1.13 | 1.11 | 1.15 |
|  | Deviation 1 | 1.14 | 1.12 | 1.16 |
|  | Deviation 2 | 1.06 | 1.04 | 1.08 |
|  |  |  |  |  |
| Schizophrenia  (SZ) | Educational achievement at age 16 | 1.17 | 1.1 | 1.24 |
|  | Deviation 1 | 1.35 | 1.28 | 1.41 |
|  | Deviation 2 | 1.25 | 1.19 | 1.31 |
|  |  |  |  |  |
| Anorexia Nervosa (AN) | Educational achievement at age 16 | 0.74 | 0.71 | 0.77 |
|  | Deviation 1 | 1.22 | 1.17 | 1.26 |
|  | Deviation 2 | 0.85 | 0.82 | 0.89 |
|  |  |  |  |  |
| Alcohol Use Disorder (AUD) | Educational achievement at age 16 | 1.93 | 1.90 | 1.96 |
|  | Deviation 1 | 1.04 | 1.02 | 1.05 |
|  | Deviation 2 | 1.26 | 1.25 | 1.28 |
|  |  |  |  |  |
| Drug Use Disorder (DUD) | Educational achievement at age 16 | 1.97 | 1.94 | 1.99 |
|  | Deviation 1 | 1.1 | 1.09 | 1.12 |
|  | Deviation 2 | 1.36 | 1.35 | 1.38 |

Figure 1: Illustrates the Hazard Ratio comparing different samples – divided by Males vs Females. The letters in the figure represent the different samples (defined in the flowchart in figure 1 of the Manuscript). Controlled for Year of Birth. * is a nominal p< 0.01 for sex differences. Power is very poor for these analyses in AN due to its rarity in males. The Y-axis represents the Hazard Ratio


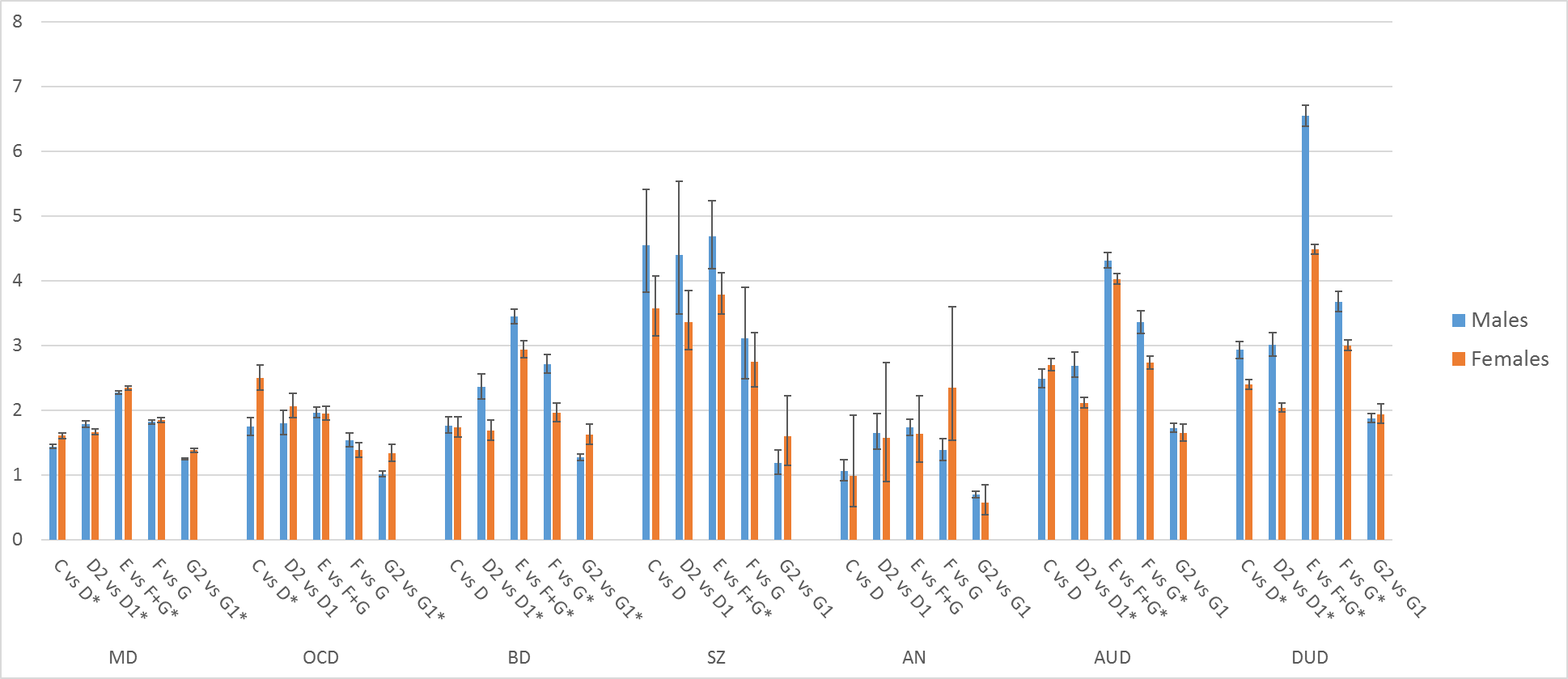


Figure 2 -- PPV values on the Y axis for Results Presented in Figure 2


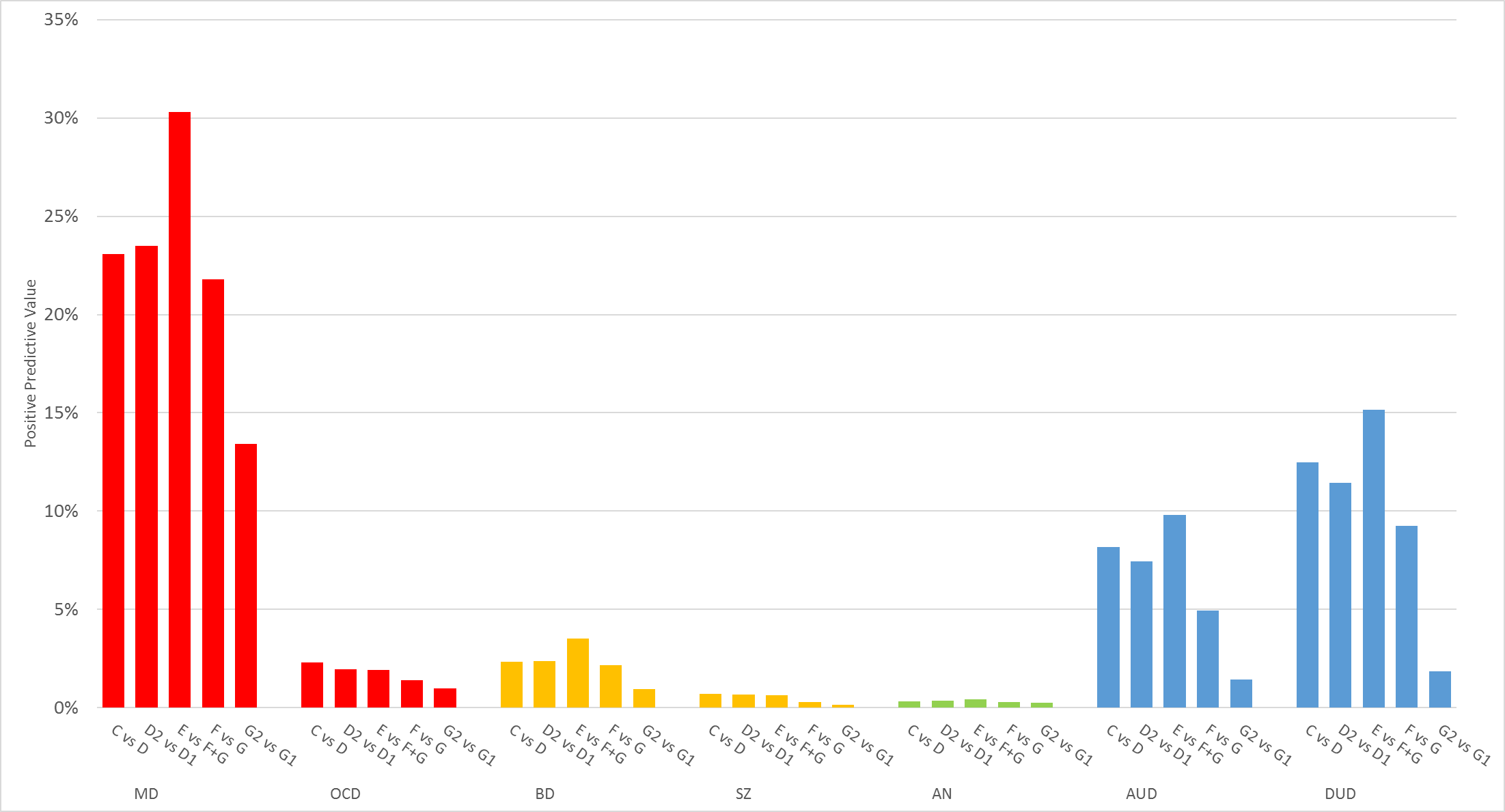


Figure 3 -- NPV values on the Y axis for Results Presented in Figure 2


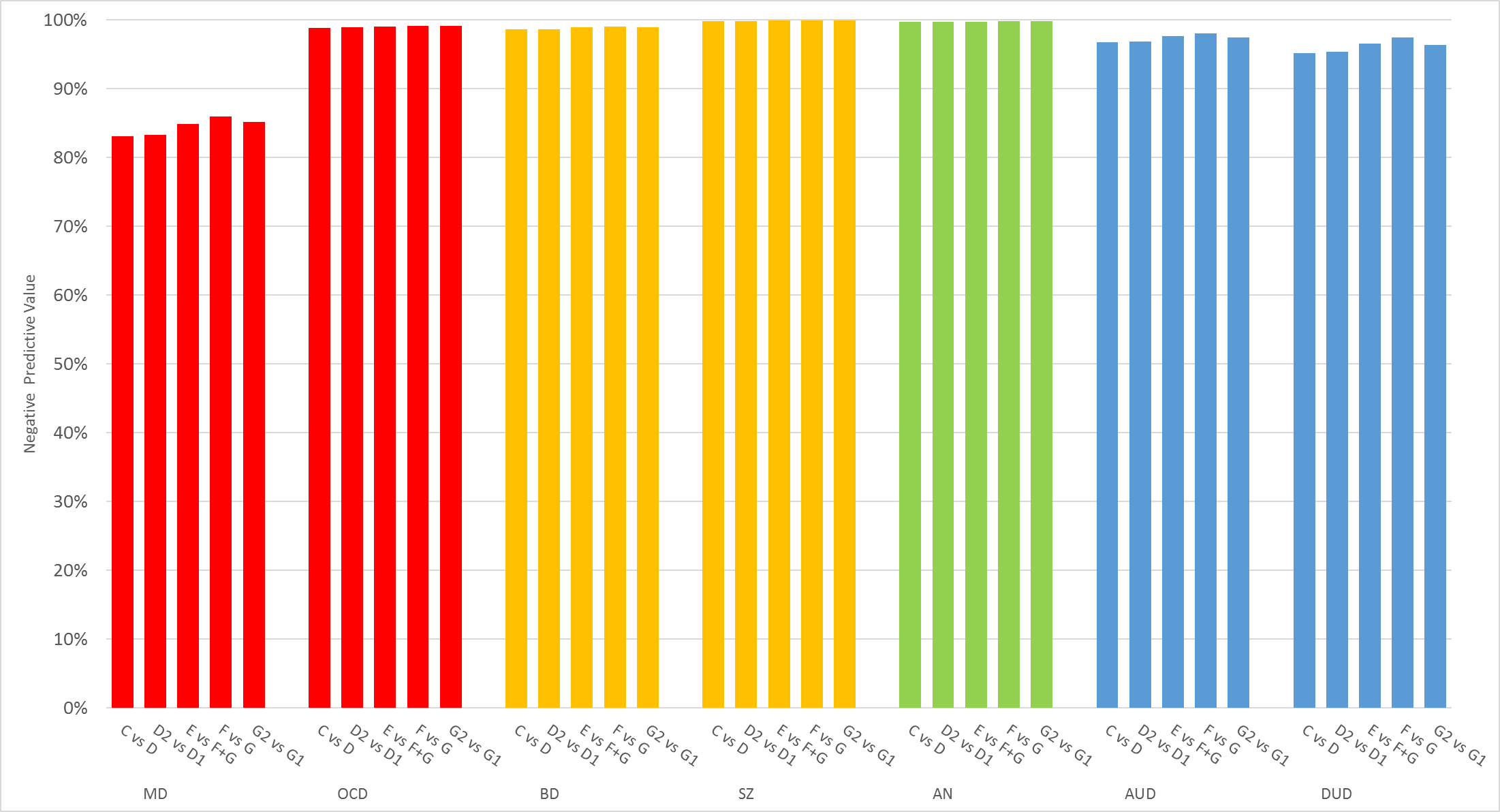


Reference List

1. Sundquist K, Malmstrom M, Johansson SE. Neighbourhood deprivation and incidence of coronary heart disease: a multilevel study of 2.6 million women and men in Sweden. *J Epidemiol Community Health.* 2004;58(1):71-77.
